# Grief and coping among relatives of patients who died of COVID-19 in intensive care during the height of the COVID-19 pandemic

**DOI:** 10.1192/bjo.2024.741

**Published:** 2024-10-15

**Authors:** Sancho Rodriguez-Villar, Elijah Oluwafemi Okegbola, Juan Arevalo-Serrano, Yasmine Duval, Annie Mathew, Carmen Rodriguez-Villar, Kirsten V. Smith, Robert Charles Kennedy, Holly G. Prigerson

**Affiliations:** Critical Care Department, King's College Hospital NHS Trust Foundation, London, UK; and GKT School of Medical Education, Faculty of Life Sciences & Medicine, King's College London, UK; St. George's Hospital Medical School, University of London, UK; Service of Internal Medicine, University Hospital Príncipe de Asturias, Spain; and Department of Medicine and Medical Specialties, Faculty of Medicine and Health Sciences, University of Alcalá, Spain; Critical Care Department, King's College Hospital NHS Trust Foundation, London, UK; Department of Haemato-Oncology, Hospital Universitario de Toledo, Spain; The Oxford Centre for Anxiety Disorders and Trauma, Department of Experimental Psychology, University of Oxford, UK; Interface Analysis Centre, University of Bristol, UK; Department of Radiology & Department of Medicine, Weill Cornell Medicine, New York, USA; and Center for Research on End-of-Life Care, Cornell University, USA

**Keywords:** Prolonged grief disorder, pathological grief, bereavement, COVID-19 pandemic, Oxford Grief-Social Disconnection Scale, Prolonged Grief Disorder Scale (PG-13-R), Quality-of-Life scale

## Abstract

**Background:**

The grief of relatives of patients who died of COVID-19 in an intensive care unit (ICU) has exacted an enormous toll worldwide.

**Aims:**

To determine the prevalence of probable prolonged grief disorder (PGD) at 12 months post-loss and beyond. We also sought to examine circumstances of the death during the COVID-19 pandemic that might pose a heightened risk of PGD, and the associations between probable PGD diagnosis, quality of life and social disconnection.

**Method:**

We conducted an observational, cross-sectional multicentre study of the next of kin of those who died of COVID-19 between March 2020 and December 2021. Participants were recruited from ICUs in South-East London. The Prolonged Grief Disorder Scale (PG-13-R), Quality-of-Life Scale (QOLS) and Oxford Grief-Social Disconnection Scale (OG-SD) were used.

**Results:**

A total of 73 relatives were recruited and assessed, all of them over a year after their loss. Twenty-five (34.2%; 95% CI 23.1–45.4%) relatives of patients who died in the ICU met the criteria for PGD. Those who met the criteria had significantly worse quality of life (QOLS score mean difference 26; 95% CI 17–34; *P* < 0.001) and endorsed greater social disconnection (OG-SD score means difference 41; 95% CI 27–54; *P* < 0.001).

**Conclusions:**

The findings suggest that rates of PGD are elevated among relatives of patients who died of COVID-19 in the ICU. This, coupled with worse quality of life and greater social disconnection experienced by those meeting the criteria, suggests the need to attend to the social deprivations and social dysfunctions of this population group.

Prolonged grief disorder (PGD) is a newly added mental disorder in the traumatic stress section of DSM-5-TR,^1^ and as such, clinical and scientific communities will need to learn how to recognise those at risk and factors that contribute to PGD onset. The DSM-5-TR criteria for PGD require that distressing symptoms of grief, including feelings of meaninglessness, emotional detachment, identity disturbances and intense loneliness, continue for at least 12 months following the loss of a close attachment. This grief response is characterised by intense longing/yearning for the deceased person and/or preoccupation with thoughts and memories of the lost person to a clinically significant (i.e. impairing) degree, nearly every day for at least the past month. PGD is associated with increased physical health problems, suicidality and functional impairment, making it essential to identify and treat cases in a timely manner.^2,3^

## Background studies

Rates of PGD range from 3 to 14% worldwide.^1,2,4,5^ Recent research found that 35% of bereaved people who lost loved ones during the COVID-19 pandemic, by any means, met the criteria for PGD at 13 months post-loss,^6^ suggesting that specific loss characteristics represent enhanced risk factors for developing a severe and enduring grief reaction. Studies directly assessing COVID-19 bereavements found higher rates of PGD symptoms than would be expected to occur in non-pandemic populations (29%).^7,8^ However, importantly, these studies were conducted in the early months following loss and did not follow up to see whether PGD symptoms were still present 12 months later. Furthermore, these studies reported general population samples with participants losing loved ones in various settings (across hospitals, care homes and in the community).

Little is known about rates of PGD among those bereaved by COVID-19 in the intensive care unit (ICU), where the sickest patients were admitted and subsequently died. Visiting restrictions were implemented in the majority of UK hospitals, to help reduce the spread of the virus and to protect visitors, in the context of a shortage of personal protective equipment (PPE) and an extreme staff workload. Many families struggled to cope with the sudden and unexpected loss of a loved one in such traumatic circumstances.

In addition to the profound sense of loss, many families reported guilt, frustration and distress at their inability to say a proper goodbye or share their last moments, because of strict hospital protocols and safety regulations.^9^ Many families were unable to have traditional funeral services to honour and remember their loved ones, because of lockdown restrictions and government guidelines.^10–12^ Relatives of patients who died of COVID-19 may have also experienced feelings of guilt, helplessness and resentment, as many were unable to visit or be with their loved ones in their final moments. Deaths in hospital/care homes increased the likelihood of poorer experiences at the end of life.^10–13^ Prior publications reported that the restrictions created by the COVID-19 pandemic represented unique risk factors that affected the grieving process for bereaved individuals, regardless of whether the death was related to COVID-19 infection.^4,14,15^

Recent studies with those bereaved during the COVID-19 pandemic have shown that social disconnection and loneliness are closely associated with grief severity.^15,16–20^ However, historically, social support has been shown to be an inconsistent predictor of grief adaptation after loss.^16–20^ Smith and Ehlers hypothesised that this discrepancy may be the result of a sense of ‘social disconnection’ experienced after bereavement, which prevents mourners from accessing available support.^21^ Social disconnection is characterised as an altered sense of social self following the loss, and is brought about by an inability to share one's grief with others for fear that it may result in negative reactions. In a recent study, it was found to predict the onset and maintenance of PGD in a community sample of bereaved adults.^22^ Given the link between loneliness and those bereaved in the COVID-19 pandemic, it is suggested that investigating the role of social disconnection as a modifiable risk factor could prove theoretically and clinically useful.

Other previous research on a specific ICU population reported that PGD appears to occur in approximately 10% of next of kin (NOK) survivors 1–2 years after the death, and is associated with accessing psychiatric services and greater dissatisfaction with ICU care.^23^ No formal specific clinical studies have been reported up to date, as far as we are aware, on patients who died of COVID-19 in the ICU during the height of the pandemic.

## Aims

The aims of the present clinical study are first, to determine the significance of PGD in the NOK of adult patients who died of COVID-19 during the two peak years of the pandemic, during ICU admission. Second, we aim to look at specific exceptional circumstances of the death that might act as risk factors: the lack of counselling or mental health support, restricted visiting and funeral ceremonies. Third, we aim to study the associations between probable PGD diagnosis, quality of life and social disconnection as modifiable factors.

## Method

### Participants

**Fig. 1:**
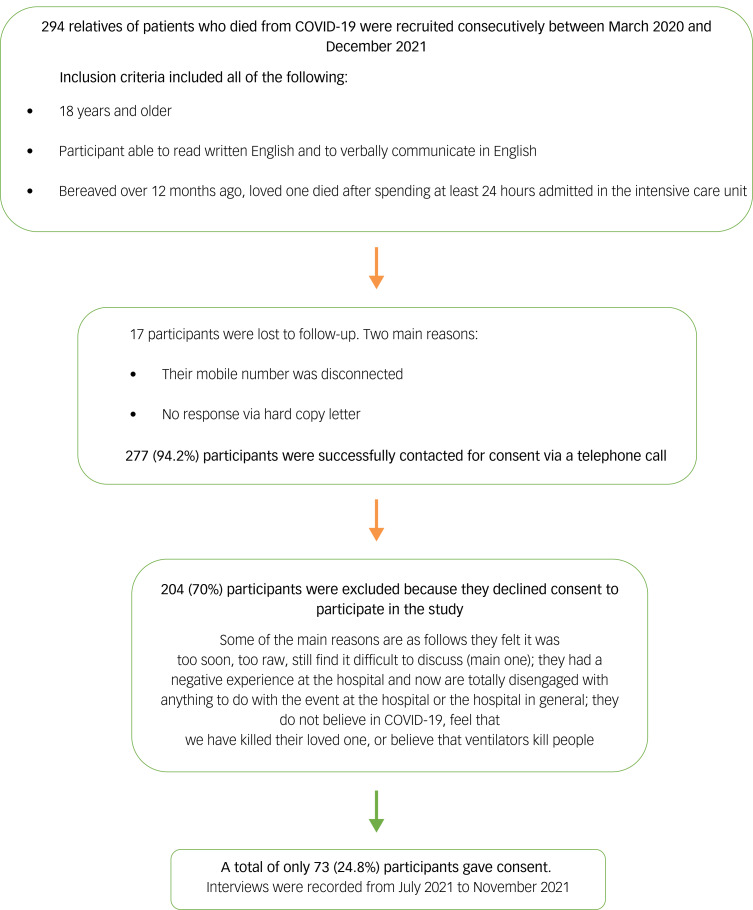
Flow diagram of cohort selection.

Members of the direct care team (also part of the research team) identified 294 eligible NOK from medical records, of whom 277 (94.2%) were contacted about the study and 73 (24.8%) gave informed verbal consent for a telephone interview, which was documented (Fig. 1). Participants were adults over the age of 18 years who were able to read and communicate in English, and whose loss occurred a minimum of 12 months prior. Participants were listed as the NOK to patients who died after spending at least 24 h in the ICU. Eligible participants were screened for deaths occurring between March 2020 and December 2021 during the height of the pandemic in all ICUs within the King's College Hospital NHS Foundation Trust and the Princess Royal University Hospital, both in South-East London, UK. A total of 73 NOK were assessed for a median of 20 months (interquartile range (IQR) 17–27) after the patient's death.

Figure 1 shows the flow diagram of cohort selection. Demographics of the deceased are presented in Table 1. Details on participant demographics, loss characteristics, end-of-life practices and support received since the loss can be found in Table 2.

**Table 1:** Demographics and other characteristics for patients who died from COVID-19

*N* (%)	73 (100)
Age (years), median (IQR)	52 (38–63)
Gender, *n* (%)	
Male	19 (26.0)
Female	54 (74.0)
Religion, *n* (%)	
Christian	28 (38.4)
Church of England	15 (20.5)
Muslin	9 (12.3)
Other	21 (28.8)
Ethnicity, *n* (%)	
White Welsh/English/Scottish/Irish/ British and other White background	36 (49.3)
Mixed/Black/Asian (e.g. Indian, Pakistani, Chinese)	13 (17.8)
Black /Asian British	10 (13.7)
Another ethnic group, e.g. Arab	14 (19.2)
Time elapsed from death to interview, months, median (IQR)	20 (17–27)
Relationship, *n* (%)	
Partner/spouse	34 (46.6)
Son/daughter	31 (42.5)
Brother/sister/niece	5 (6.8)
Mother/cousin/friend	3 (4.1)
Support given (between the loss and our study contact), *n* (%)	47 (64.4)
Any counselling since the death, *n* (%)	36 (49.3)
Any mental health condition/depression, *n* (%)	23 (31.5)
Able to visit, *n* (%)	55 (75.3)
Any video calls, *n* (%)	45 (61.6)
Updates from the medical team, *n* (%)	72 (98.6)
Other family member/friend died of COVID-19, *n* (%)	27 (37.0)
Funeral/religious ceremonies affected in anyway by the pandemic/lockdown, *n* (%)	71 (97.3)
Vaccine status, *n* (%)	
Vaccinated	3 (4.1)
Unvaccinated	1 (1.4)
Not applicable	69 (94.5)
PG-13-R score, median (IQR)	24 (19–37)
Prolonged grief disorder (PG-13-R score >30), *n* (%)	25 (34.2)
PG-13-R Question 13 ‘Have the symptoms above caused significant impairment in social, occupational or other important areas of functioning?’, *n* (%)	33 (45.2)
OG-SD score, median (IQR)	22 (15–65)
QOLS score, median (IQR)	82 (72–91)

Ethnic group categories derived from the National Health Service equal opportunities monitoring form. IQR, interquartile range; PG-13-R, Prolonged Grief Disorder Scale; OG-SD, Oxford Grief-Social Disconnection Scale; QOLS, Quality-of-Life Scale.

**Table 2:** Patient demographics and other characteristics for relatives of patients who died from COVID-19

*N* (%)	73 (100)
Age (years), median (IQR)	66 (58–73)
Gender, *n* (%)	
Male	52 (71.2)
Female	21 (28.8)
Religion, *n* (%)	
Christian/Catholic	28 (38.4)
Church of England	15 (20.5)
Muslim	9 (12.3)
Others	21 (28.8)
Children, median (IQR)	2 (1–3)
Occupation/employment, *n* (%)	
Active	39 (54.2)
Retired	33 (45.8)
Main provider of income, *n* (%)	23 (31.5)
Carer for anyone, *n* (%)	11 (15.1)

IQR, interquartile range.

### Public involvement

Public involvement in research means research that is conducted ‘with’ or ‘by’ the public, not ‘to’, ‘for’ or ‘about’ them. It means that patients or other people with relevant experience contribute to how research is designed, conducted and disseminated.^24^ To understand if participants from this population would be interested in taking part in the research, the principal investigator conducted patient and public involvement interviews with two different families of patients who died of COVID-19 in the ICU, after verbal consent. The relatives expressed the desire for an interview without a video call, using standard telephone communication, and to be notified beforehand of the time of day and time. Both relatives reported that they were supportive of the research protocol and the design of the study.

### Ethical approval

The study conformed to the Declaration of Helsinki and to local applicable regulatory provisions. The study was approved by the local Research and Development (R&D) Department, reference KCHN/A; the Research Ethics Committee (REC), reference 22/YH/0045; and the Regional Ethics Committee Yorkshire & The Humber – South Yorkshire Research Ethics Committee.

### Measures

#### The Prolonged Grief Disorder Scale

The Prolonged Grief Disorder Scale (PG-13-R) is a 13-item self-report measure assessing the prevalence and severity of PGD symptoms (e.g. yearning, disbelief, feelings of meaninglessness and identity confusion).^1^ The measure is a validated tool to identify individuals who meet the criteria for PGD according to the DSM-5-TR.^1,25–28^ Caseness of PGD was shown to have high rates of diagnostic correspondence, with a score of >30 on items 3–12 of the measure.^1^ Internal consistency in our sample using the PG-13-R symptoms items 3–12 cohered well (Cronbach's alpha 0.92).

#### The Oxford Grief-Social Disconnection Scale

The Oxford Grief-Social Disconnection Scale (OG-SD) is a 15-item self-report questionnaire assessing different aspects of social disconnection following bereavement.^22^ A negative interpretation of others’ reactions to grief expression (e.g. ‘Others will not be able to manage if I tell them how I feel about the loss’), an altered sense of self (e.g. ‘I can't be myself around other people the way I used to’) and belief that there is safety in solitude (e.g. ‘It is easier to be alone than to have to pretend to feel ok’). These feelings were shown to predict the development and maintenance of psychological distress.^17^ Participants are asked to rate the extent to which they agreed with each statement, on a seven-point scale (1, totally disagree; 7, totally agree) in the past month. Cronbach's alpha of the 15 items on the OG-SD scale was 0.99.

#### The Quality-Of-Life Scale

The Quality-of-Life Scale (QOLS) consists of 16 items that measure an individual's overall sense of well-being. Each item is rated on a seven-point Likert scale, ranging from 1 (strongly disagree) to 7 (strongly agree). Scoring for the QOLS is simple. For each item, total the responses from all 16 items to obtain the total QOLS score. Higher scores indicate a higher quality of life. The total score can range from 16 to 112. The QOLS is a reliable and valid instrument for measuring quality of life from the perspective of the respondent.^29^ It focuses on domains that come from the qualitative descriptions of a wide range of adults across gender, cultural and language groups.^29^ Cronbach's alpha was 0.94 for the 16 items on the QOLS scale. All three instruments demonstrated a high degree of internal consistency.

Although we have a standardised set of questions/answers, we also allowed the participants to elaborate and speak freely to us about their current state of grief, hospital experience and pandemic experiences, which we gathered information from to explore risk factors associated with PGD.

### Procedure

This was an observational, cross-sectional and multicentre study. Local clinical investigators screened eligible patients from all ICUs of hospitals affiliated with the King's College NHS Foundation Trust in London, England.

Eligible participants were invited to a telephone interview with a trained interviewer who administered the measures and collected data on end-of-life care experiences, funeral disruptions, a previous history of mental health problems and bereavement support received, if any. Interviews conformed to ethical guidelines on conducting remote research with vulnerable populations. An individual interview was conducted with the NOK at a median of 20 months after the loss (IQR 17–27). Participants were offered a check-in call 24 h after research participation, to screen for elevated distress resulting from the research process.^30^ However, many participants preferred text rather than telephone communication, which was especially the case for older study participants.

Those NOK who reported during interview that they were assessed recently for severe depression or who had attempted suicide in the previous 3 months and scored high for PGD were referred to the South London and Maudsley NHS Foundation Trust (SLaM), which specialises in mental health. Participants were not excluded from further participation in the study based on psychiatric status, and their status was also analysed statistically.

Based on the threshold value, the NOK identified as having PGD were informed and offered direct referral to their general practice. They were also provided contact details for local mental healthcare support services, counselling and the hospital bereavement team.

### Data analysis

Values were reported as absolute and relative (percentages) frequencies for categorical data, and by continuous variables as medians and IQRs. Associations between variables and PGD were estimated. The proportion of individuals with probable PGD in each group (PGD and the other binary predictors such as gender, previous psychiatric history, etc.) were calculated for binary predictor with the chi-squared test or Fisher exact test (applied to the relationship between probable PGD diagnosis and gender of the participant, previous history of psychiatric illness and funeral delay; applied to the relationship between the participant wanting to say goodbye and the variable of being present, in person or through video call, with the patient in the days before their death). The proportions with probable PGD in each category were calculated for categorical predictor variables by chi-squared test or Fisher exact test (applied to investigate the relationship between a probable PGD diagnosis and relationship to the deceased, education, religion and ethnicity, introducing them as dummy variables). Continuous data were summarised using median and IQR, and analysed using the Student's *t*-test or non-parametric Mann–Whitney-*U* test, which was applied to the relationship between age and probable PGD diagnosis. Backward stepwise predictive logistic regression modelling (multivariate predictive model) was used to investigate the most informative influences on risk of a probable PGD diagnosis. Starting with the initial maximum model with the predictors of PGD, the order of selection to evaluate the exclusion of the predictors was by descending statistical significance until all of the predictors in the final model had a *P* < 0.05.

### Sample size calculation

Assuming a population size of 1000 people, 10% as an estimated proportion of PGD, a confidence interval of 95%, a design effect of 1 and a precision of 7%, we required 66 participants for our study.^31^

## Results

### Initial descriptive statistics

Table 1 shows the demographics and other characteristics of NOK. In 31 (42.4%) cases, the NOK was the deceased's son or daughter; in 34 (46.6%) cases, the NOK was the partner/spouse; in five (6.8%) cases, the NOK was a brother/sister/niece and in three (4.1%) cases, the NOK was a mother/cousin/friend. Table 2 shows the demographics and other characteristics of deceased patients.

### Prevalence of PGD and its relationship with possible risk factors

Twenty-five (34.2%; 95% CI 23.1–45.4%) NOK of patients who died in the ICU met the criteria for PGD. Table 3 shows patient and NOK demographics and other characteristics, by PGD (PG-13-R score >30).

**Table 3:** Patient and next of kin demographics and other characteristics, by presence of prolonged grief disorder

	PGD	No PGD	Relative risk/mean difference^a^	*P-*value
	(PG-13-R score >30)	(PG-13-R score ≤30)		
	*n* (%)/median (IQR)^b^	*n* (%)/median (IQR)^b^	(95% CI)	
*N*	25	48		
Patient				
Age (years)	62 (57–67)	69 (59–77)	5.6 (0.1–11.2)	0.046
Gender			1.27 (0.60–2.75)	>0.200
Male	19 (76.0)	33 (68.8)		
Female	6 (24.0)	15 (31.3)		
Religion				>0.200
Christian	8 (32.0)	20 (41.7)		
Church of England	4 (16.0)	11 (22.9)		
Muslin	4 (16.0)	5 (10.4)		
Other	9 (36.0)	12 (25.0)		
Children	2 (2–3)	2 (1–2)	0.31 (−0.31 to 0.93)	0.164
Occupation			1.69 (0.83–3.44)	0.132
Active	16 (66.7)	23 (47.9)		
Retired	8 (33.3)	25 (52.1)		
Main source of income *‘*the main breadwinner*’*	13 (52.0)	10 (20.8)	2.36 (1.28–4.33)	0.007
Carer for anyone	5 (20.0)	6 (12.5)	1.41 (0.67–2.96)	>0.200
Next of kin				
Age (years)	56 (44–62)	50 (38–65)	2.12 (−5.24 to 9.48)	>0.200
Gender			0.54 (0.21–1.38)	0.159
Male	4 (16.0)	15 (31.3)		
Female	21 (84.0)	33 (88.8)		
Religion				>0.200
Christian	9 (36.0)	19 (39.6)		
Church of England	3 (12.0)	12 (25.0)		
Muslim	4 (16.0)	5 (10.4)		
Other	9 (36.0)	12 (25.0)		
Ethnicity				>0.200
White British	12 (48.0)	24 (50.0)		
Black	6 (24.0)	7 (14.6)		
Black British	2 (8.0)	8 (16.7)		
Other	5 (20.0)	9 (18.8)		
Time from death to interview (months)	21 (18–27)	20 (17–26)	1.78 (−1.49 to 5.04)	>0.200
Relationship			2.95 (1.40–6.20)	0.002
Partner/spouse	18 (72.0)	16 (33.3)		
Other	7 (28.0)	32 (66.7)		
Support given (general practitioner/social services)	22 (88.0)	25 (52.1)	4.06 (1.34–12.27)	0.002
Any counselling since the loss (psychological or psychiatric)	18 (72.0)	18 (37.5)	2.64 (1.25–5.55)	0.005
Any past mental health condition/depression (self-reported)	13 (52.0)	10 (20.8)	2.36 (1.28–4.33)	0.007
Able to visit loved one before their death	22 (88.0)	33 (68.8)	2.40 (0.81–7.09)	0.070
Any video calls	13 (52.0)	32 (66.7)	0.67 (0.36–1.26)	>0.200
Updates from the medical team	24 (96.0)	48 (100)		>0.200
Other family member/friend died of COVID-19	10 (40.0)	17 (35.4)	1.14 (0.60–2.16)	>0.200
Funeral/religious ceremonies affected by the pandemic/lockdown	24 (96.0)	47 (97.9)	0.68 (0.16–2.81)	>0.200
Vaccine status				>0.200
Vaccinated	0 (0.0)	3 (6.3)		
Unvaccinated	1 (4.0)	0 (0.0)		
Not applicable	24 (96.0)	45 (93.8)		
PG-13-R score	41 (37–46)	20 (17–24)	21.51 (18.91–24.11)	<0.001
Question 13. ‘Have the symptoms above caused significant impairment in social, occupational or other important areas of functioning?’	22 (88.0)	11 (22.9)	8.59 (2.92–27.10)	<0.001
OG-SD score	68 (30–93)	19 (15–24)	41 (27–54)	<0.001
QOLS score	68 (46–78)	90 (79–99)	26 (17–34)	<0.001

First objective: PG-13-R score and question 13. Second objective: PGD predicted variables. Third objective: OG-SD score and QOLS score. PGD, prolonged grief disorder; PG-13-R, Prolonged Grief Disorder Scale; IQR, interquartile range; OG-SD, Oxford Grief-Social Disconnection Scale; QOLS, Quality-of-Life Scale.

a. Relative risk for categorical data and mean difference for quantitative data.

b. *n* (%) for categorical data and median (IQR) for quantitative data.

The univariate analysis of each predictor with the PGD response showed that the strongest associations were with kinship relationship to the deceased (odds ratio 2.95; 95% CI 1.40–6.20; *P* = 0.002); those who had lost a partner/spouse were almost three times more likely to be in the PGD group that those with any other type of relationship. The same occurred for those who had received psychiatrist support (odds ratio 2.95; 95% CI 1.40–6.20; *P* = 0.002), any previous counselling since the death (odds ratio 2.64; 95% CI 1.25–5.55; *P* = 0.005) or had history of any mental health condition/depression (odds ratio 2.36; 95% CI 1.28–4.33; *P* = 0.007), who were more than two times more likely to be in the PGD group.

### Relationship between PGD, OG-SD score and quality of life

All 15 items of the OG-SD score were significantly associated with PGD, at *P* < 0.001 for each item.

With the limitation of only 25 events (PGD), the initial maximum model has the four variables with the lowest *P*-value (relationship, support given, OG-SD score and any mental health condition/depression). The multivariate model is based on an initial model with four predictors that are more than the 20 events allowed (one predictor for every ten events or non-events, whichever is less). By backward regression, two predictors whose explanatory contribution is scarce were eliminated, and the final model is left with the two predictors shown in Table 4.

**Table 4:** Estimation of multivariate model of the outcome presence of prolonged grief disorder

Global model		Nagelkerke *R*^2^ 0.55	*P* < 0.001
Variables	OG-SD score	Odds ratio 1.06 (95% CI 1.03–1.08)	*P* < 0.001
	Relationship of next of kin	Odds ratio 3.97 (95% CI 1.03–15.37)	*P* = 0.046

OG-SD, Oxford Grief-Social Disconnection Scale.

The final model is shown in Table 4 (*P* < 0.001) and has two variables (relationship and OG-SD score); it explains 55% of the total variability of PGD (Nagelkerke *R*^2^ of 0.55).

### Predictive multivariate analysis with logistic regression

**Fig. 2:**
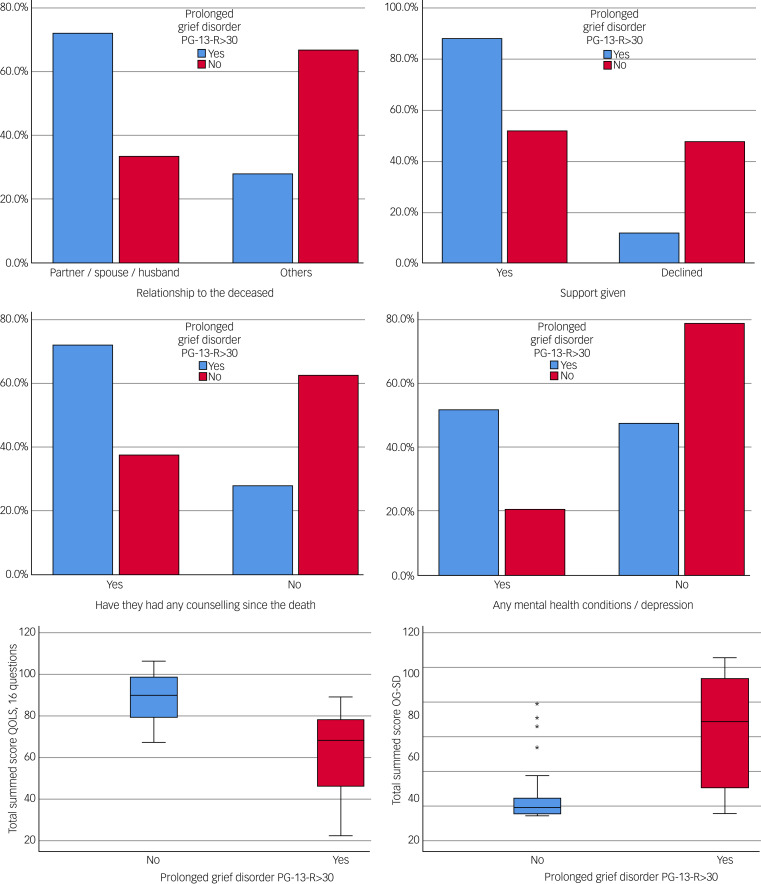
Barr plots (of relationship, support given, any counselling since the death and any mental health condition/depression), and boxplots (of QOLS and OG-SD scores) of PGD (PG-13-R score > 30). OG-SD, Oxford Grief-Social Disconnection Scale; PG-13-R, Prolonged Grief Disorder Scale; QOLS, Quality-of-Life Scale.

Those with PGD had a QOLS score of 68 (IQR 46–78) points, significantly lower than the average score of those without PGD (90 points; IQR 79–99; mean difference of 26; 95% CI 17–34 points; *P* < 0.001). Those with PGD had an average OG-SD score of 41 points, significantly higher than the average score of those without PGD. For a very high percentage of participants, the quality of life was reduced (QOLS mean difference 26 points; 95% CI 17–34; *P* < 0.001) and isolation was increased (OG-SD mean difference 41 points; 95% CI 27–54; *P* < 0.001). There was a clear relationship between these scores and the presence of PGD. A poor score on the QOLS was associated with the presence of PGD, as was a high score on the OG-SD. The plot is shown in Fig. 2.

## Discussion

Our results show that 34% of relatives of patients who died from COVID-19 in the ICU met probable diagnoses for PGD. Prior ICU research has found a 4–10% prevalence of PGD at 1–2 years after the death of a relative with a high mortality score on admission.^23^ A systematic review and meta-analysis of the overall prevalence of PGD suggests that one out of ten bereaved adults exposed to non-violent bereavement is at risk for PGD. ^32^ Another study found that of the 38% who were ICU bereaved at 6 months, 42% had ‘complicated grief’ (another name for PGD).^33^ Other studies have found a 52% occurrence in relatives following the death of a relative in the general ICU at 6 and 12 months post-death.^34^

However, undergoing a recent loss during the pandemic resulted in higher grief levels than undergoing a recent loss before the pandemic.^35^ Other research has also found that there are higher levels of acute grief among individuals bereaved as a result of COVID-19 compared with people bereaved as a result of natural loss.^10^

In our study, the emotional pain, and more specifically, the anger and bitterness (recorded in item 8 of the PG-13-R), were very high. Participants felt anger toward the virus, as they felt that the virus stole their loved one, and if the virus was not around, they would still be there. They said if they died from cancer, stroke, heart attack, etc., it would be easier to accept. However, the virus seems like an external element that stole their loved one: again, the thoughts of more ‘unnatural death’ are less acceptable. Some were also very angry at people who did not follow the lockdown restrictions.^36^ A study in hospital settings published in 2022 reported symptoms PGD were above the cut-off in 1.3% in the non-COVID-19-group and 48.6% in the COVID-19 group, but this is difficult to interpret as the study has not had an appropriate follow-up time to assess prolonged grief from the time of death. The follow-up period was <6 months, so it is likely to overestimate the prevalence, among other limitations. The study period overlaps with the start of the pandemic, whereas our study is focused on the height of the pandemic; therefore, it is unlikely to take fully into account all potential risks associated. Also, it used a self-questionnaire instead of a clinical interview.^37^

In a Canadian matched cohort study in a hospital setting in 2022, almost a third of family members who experienced bereavement during the early stages of the COVID-19 pandemic had symptoms of severe grief, regardless of the cause of death.^38^ This prevalence is similar to the results in our work as well.

A previous systematic review of the literature on predictors of complicated grief recognised risk factors for poor bereavement outcomes, such as age, gender, relationship to the deceased and expectedness of the death.^36^ Our results were significant for the relationship to the deceased (e.g. partner or spouse) and the expectedness of the death; both can be closely related to the circumstances of the pandemic. Also, it is worth mentioning that being the main source of income for a family (either as a partner or spouse) and the lack of psychological support are also risk factors in our study.

Some NOK ‘felt a lot of guilt’ because they felt that they might have spread the virus to their NOK and in doing so they feel that they were the cause of their death. There was also a lot of guilt that they ‘survived COVID-19’ after having been sick with it themselves, although not near to death, whereas their loved one did not survive. This was revealed to us by being very frequently mentioned during the interviews.^22,39^

It has been suggested that circumstances surrounding death from COVID-19, or living during the pandemic, may affect the normal grieving process. Examples of this are experiencing multiple deaths, lack of social support because of social distancing and inability to perform typical grief rituals because of restrictions placed on funerals.^40^ In our study, the hospital restrictions during the pandemic affected NOK psychologically in different ways. First, they felt their loved one ‘died alone in hospital’. With respect to this, the presence of the medical team tending to their relatives was not comforting to them. They wanted to be present during the final moments of their NOK, and not being able to do this still haunts them. Some of the exceptional instances of NOK who were able to say goodbye in the hospital said that this gave them some sense of closure, which helped them process their grief, as they could say loving and personal messages to their NOK when they were dying – this has helped them to process the death. Second, because NOK were not able to see the clinical deterioration through the patient's ICU admission because visiting was not allowed, there also is a disconnection from accepting the health status of their loved one who was, in their eyes, sick but not near death when they last saw them on admission. Not seeing the loved one's decline for themselves has made accepting the death harder. Therefore, many NOK stated that their loved one was stable for days, then they received a sudden telephone call that their loved one was imminently dying, and they had to rush to the hospital. This is something that was also very frustrating for them, as they could not understand the rapid decline from the ‘stable’ updates and/or felt that maybe they were not given a fully informed update about the status of their NOK. It was a difficult process to believe without being physically present.

Finally, the third point related to the hospital and government restriction policies when NOK had previously had COVID-19. They were frustrated as they felt that they had already been exposed, so the risk to them was minimal, and so they did not understand why they could not visit during their dying moments. With others, there were frustrations because they were willing to comply with wearing PPE to see their loved one, but were still not allowed to come in to see them in the hospital; some people voiced their frustrations when the UK lockdown was lifted, or seeing people violate lockdown rules but they could still not come into the hospital to see their loved one. The restrictions on the number of people who could attend were difficult, especially for large Irish or Muslim families. Religious ceremonies, such as the ‘Nine-Night’, also known as ‘Dead Yard’, are a funerary tradition originating in Africa and practised in Caribbean countries, but this was unable to be performed. One family member wanted to bring their father's body back to their original country, as that was always his dying wish, but they were unable to do so because of governmental restrictions.

One participant described how her husband had to be cremated (the very first few deaths of the first wave), and he was very afraid of being ‘burnt alive’. The fact that this was the only option that the government would allow was very distressing to her even nearly 3 years later. The hospital and ICU restrictions at the time of the pandemic had a great impact. There was a lot of frustration toward the strict visiting policy, which seemed to be one of the most long-standing issues that contributes to their grief.

Social disconnection played a key role in grief. Those who endorse social disconnection report concern that they will be judged negatively if they were to share their feelings with others, an altered sense of self in social situations and a belief that they can only truly be authentic in their grief when alone. Results demonstrated it as a predictor of concurrent and prospective PGD symptoms.^22^ In a separate study looking at 647 individuals bereaved at least 6 months prior, social disconnection was found not only to significantly predict those who met criteria for prolonged grief and post-traumatic stress disorder, but also increased the likelihood of having both conditions compared with individuals allocated to only one of the symptom areas.^22,40^

A non-clinical study in a Chinese population, using an online survey in September 2020, where 476 participants were recruited through social network websites (e.g. Baidu, WeiBo) and mobile applications (e.g. WeChat), from which we ignore the number of individuals approached, also suggests that the prevalence of PGD in people bereaved as a result of COVID-19 was high (37.8%). The study had very similar results to our study, but other than that, little is known about the prevalence and symptom severity of PGD among people whose loved ones died from the COVID-19 pandemic.^8^ There are currently no other studies available to compare directly, and we believe this may be attributable to the chronological order in which the countries were affected around the world, among other reasons.

The risk of developing PGD should be identified, and bereavement support should be provided as soon as possible.^8^ The relationship between PGD and quality of life is complex. PGD is associated with poorer physical and psychological health, poorer social and occupational functioning, and more significant emotional distress. In addition, people with PGD are more likely to experience a decrease in overall quality of life. They may have difficulty engaging in meaningful activities, forming and maintaining relationships and regulating their emotions. In our study, the NOK mainly felt that their friend/partner who died was ‘their person’, and now that they were gone, they just wanted to be on their own and no longer looked for new friendships/relationships. In a recent study, the results showed that PGD symptoms of meaninglessness and role confusion were linked with reduced psychological quality of life, trust difficulties were linked with reduced social quality of life and bitterness was linked with reduced environmental quality of life.^41^

These effects can lead to a feeling of hopelessness and a decrease in quality of life. At the same time, research suggests that interventions that target PGD may improve quality of life. Such interventions include cognitive–behavioural therapy, exposure therapy and supportive psychotherapy. In a previous publication, the following themes were addressed: harmonisation in the probable diagnosis of PGD, screening tools and interventions, pharmacotherapy, special attention for the elderly, special attention for children and adolescents, and a causal system perspective for understanding grief and PGD.^42^

### Strengths and limitations

As far as we are aware, this is the first formal clinical study to estimate specifically the prevalence of PGD in the NOK where the deceased died directly from COVID-19 in the ICU. All cases were during the peak 2 years of the pandemic and a year after the deceased died. This study was designed and developed by clinicians and researchers, using validated diagnostic tools through a well-structured clinical interview conducted by trained interviewers. The study protocol was comprehensively examined by an ethics committee, and a previous public inquiry was conducted. The results indicate that PGD was present in over a third of COVID-19-related bereaved individuals during the height of the pandemic; this was of the bereaved population studied in a UK sample.

Our study had some strengths in identifying the prevalence of PGD during the COVID-19 pandemic compared with previous publications,^9–11^ like having an ethics/institutional review board, individuals who were actively recruited, knowing the total number of approached individuals, knowing the percentage of individuals who declined participation, an appropriate follow-up period to assess PGD from the time of death, no use of online surveys and a direct contact with clinical experts. Also, we did not use untraceable online databases and sources such social networks or mobile applications, and obtained data on basic demographic characteristics.

With respect to the limitations of the study, the number of people who declined interviews was high (around 70%). We suspect that selection bias may have resulted in the more distressed individuals being less likely to participate, based on the comments made by the patients, such as ‘I am still not able to talk about it’ or ‘It is too distressing’, making the prevalence rates conservative, and it is likely that the rate of PGD was underestimated as a result. This response can be attributed to several factors that were collected at the time the informed consent was asked. First, emotional distress, as the grief and sorrow associated with loss can be overwhelming and make it difficult for relatives to engage in activities that could remind them of their pain, such as participating in the study. Second, privacy and sensitivity, as some family members felt that participating could expose personal and sensitive information about their deceased loved one or themselves. Third, time and energy constraints, as dealing with a loss can be a draining process, both physically and emotionally. Fourth, distrust or scepticism, as in some cases, relatives might harbour feelings of distrust toward institutions or researchers, especially if they believe that the interview could be intrusive or misrepresent their loved one's experience. We are not able to compare our results to any of the studies currently published, as none of them recorded the total number of the NOK approached and the percentage of individuals who declined participation. More research has been done on PGD and end-of-life care or in bereaved populations of people in general, in which a prevalence rate of 3% is expected.^1^

Finally, the study used multivariate analysis with the backward regression procedure, which has its limitations, especially with the few predictors that can be introduced into the model, considering the few events.

### Implications for future research

A focus for further research in the future would be subsyndromal symptoms, which are typically ongoing, rarely resolve spontaneously and pose a risk for the emergence of, or transition to, PGD itself; in addition to frequently co-occurring disorders, such as major depression or post-traumatic stress disorder, with definite implications for clinical practice.

In conclusion, the findings of our study suggest that rates of PGD were elevated among relatives of patients who died of COVID-19 in the ICU compared with relatives of patients who died from other natural causes. This, coupled with worse quality of life and greater social disconnection experienced by those meeting the criteria, suggests the need to attend to the social deprivations and social dysfunctions that were exacerbated amid the global pandemic and undermine adjustment to loss. Interventions to reduce PGD that target filling the social void created by a significant interpersonal loss might hold promise for reducing prolonging intense, disabling and distressing symptoms of grief in bereaved relatives.

## Data Availability

The data that support the findings of this study are available on request from the corresponding author, S.R.V. The data are not publicly available due to privacy/ethical restrictions.
